# Role and Potential Mechanism of Heme Oxygenase-1 in Intestinal Ischemia-Reperfusion Injury

**DOI:** 10.3390/antiox11030559

**Published:** 2022-03-15

**Authors:** Kazuhiro Katada, Tomohisa Takagi, Takaya Iida, Tomohiro Ueda, Katsura Mizushima, Akifumi Fukui, Tetsuya Okayama, Kazuhiro Kamada, Kazuhiko Uchiyama, Takeshi Ishikawa, Yuji Naito, Yoshito Itoh

**Affiliations:** 1 Molecular Gastroenterology and Hepatology, Graduate School of Medical Science, Kyoto Prefectural University of Medicine, Kyoto 602-8566, Japan; katada@koto.kpu-m.ac.jp (K.K.); takayaii@koto.kpu-m.ac.jp (T.I.); tm-ueda@koto.kpu-m.ac.jp (T.U.); mizusima@koto.kpu-m.ac.jp (K.M.); afukui@koto.kpu-m.ac.jp (A.F.); t-oka@koto.kpu-m.ac.jp (T.O.); k-kamada@koto.kpu-m.ac.jp (K.K.); k-uchi@koto.kpu-m.ac.jp (K.U.); iskw-t@koto.kpu-m.ac.jp (T.I.); yitoh@koto.kpu-m.ac.jp (Y.I.); 2 Department for Medical Innovation and Translational Medical Science, Graduate School of Medical Science, Kyoto Prefectural University of Medicine, Kyoto 602-8566, Japan; 3 Department of Human Immunology and Nutrition Science, Graduate School of Medical Science, Kyoto Prefectural University of Medicine, Kyoto 602-8566, Japan; ynaito@koto.kpu-m.ac.jp

**Keywords:** ischemia-reperfusion injury, heme oxygenase-1, BTB and CNC homolog 1, NF-E2-related factor 2, carbon monoxide, nuclear factor-κB, neutrophil-endothelial interaction

## Abstract

Intestinal ischemia-reperfusion (IR) injury is a complex, multifactorial, and pathophysiological condition with high morbidity and mortality, leading to serious difficulties in treatment, especially in humans. Heme oxygenase (HO) is the rate-limiting enzyme involved in heme catabolism. HO-1 (an inducible form) confers cytoprotection by inhibiting inflammation and oxidation. Furthermore, nuclear factor-erythroid 2-related factor 2 (Nrf2) positively regulates HO-1 transcription, whereas BTB and CNC homolog 1 (Bach1) competes with Nrf2 and represses its transcription. We investigated the role and potential mechanism of action of HO-1 in intestinal IR injury. Intestinal ischemia was induced for 45 min followed by 4 h of reperfusion in wild-type, Bach1-deficient, and Nrf2-deficient mice, and a carbon monoxide (CO)-releasing molecule (CORM)-3 was administered. An increase in inflammatory marker levels, nuclear factor-κB (NF-κB) activation, and morphological impairments were observed in the IR-induced intestines of wild-type mice. These inflammatory changes were significantly attenuated in Bach1-deficient mice or those treated with CORM-3, and significantly exacerbated in Nrf2-deficient mice. Treatment with an HO-1 inhibitor reversed this attenuation in IR-induced Bach1-deficient mice. Bach1 deficiency and treatment with CORM-3 resulted in the downregulation of NF-κB activation and suppression of adhesion molecules. Together, Bach1, Nrf2, and CO are valuable therapeutic targets for intestinal IR injury.

## 1. Introduction

Intestinal ischemia-reperfusion (IR) injury, a life-threatening abdominal emergency condition, develops in multiple clinical settings, including mesenteric artery occlusion, mesenteric venous thrombosis, major cardiovascular surgery, trauma, shock, and small-intestinal transplantation, and shows high morbidity and mortality [1,2]. Early diagnosis and treatment are required to prevent bowel infarction. However, existing circumstances have not yet led to dramatic changes in human treatment strategies. Therefore, further investigation of new therapeutic targets for intestinal IR injury, even in animals, is required.

Intestinal IR injury causes a molecular and cellular inflammatory response within the intestine, including increased oxidative stress, activation of inflammation-relevant transcription factors, such as nuclear factor (NF)-κB, and recruitment of polymorphonuclear leukocytes (PMNs) [3,4,5,6]. The activation and recruitment of PMNs are rate-limiting events in the pathogenesis of IR injury [7,8]. It is important to note that an increase in PMN accumulation in the IR-induced intestine results in further intestinal tissue damage and often bacterial translocation and subsequent systemic complications leading to multiple organ failure [3,9]. Therapeutic targets should be considered based on the pathophysiology of the intestinal IR injury. The attenuation of oxidative stress and leukocyte involvement is considered to be the most important target for the protection against intestinal IR injury.

Heme oxygenase (HO) is a rate-limiting enzyme that catalyzes the degradation of heme to biliverdin, free iron, and carbon monoxide (CO) [10,11,12]. HO-1 is one of three mammalian HO isozymes and is a stress-responsive protein induced by various stimuli, including oxidative stress, IR, heavy metals, cytokines, and heme (its substrate). HO-1 expression is regulated by various transcriptional proteins, including the heat shock factor-1 (HSF1), NF-κB, activator protein-1 (AP-1), nuclear factor-erythroid 2-related factor 2 (Nrf2), and the BTB and CNC homolog 1 (Bach1). Interestingly, Nrf2 positively regulates HO-1 transcription. In contrast, Bach1 competes with Nrf2 and suppresses its transcription [10,13,14]. Therefore, there may be various novel therapeutic targets for the regulation of HO-1 expression.

We previously reviewed the therapeutic roles of CO in intestinal IR injury and its cell-specific anti-inflammatory effects [15], concluding that HO-1-derived endogenous and exogenous CO can confer anti-inflammatory and cytoprotective effects in IR-induced intestinal injury. However, CO delivery systems are not sufficient for delivering CO with high safety and efficiency. Therefore, new CO delivery systems, including CO-releasing molecules (CORMs), are required.

In the present study, we focused on Bach1, Nrf2, and CO (CORM-3) as therapeutic targets to regulate HO-1 expression and investigated the role and potential mechanism of HO-1 in intestinal IR injury using Bach1-deficient and Nrf2-deficient mice and the administration of CO (CORM-3).

## 2. Materials and Methods

### 2.1. Animals

Seven-week-old male wild-type (WT) mice (C57BL/6J mice) were purchased from Shimizu Laboratory Supplies (Kyoto, Japan). Bach1-deficient mice were gifted by Prof. Kazuhiko Igarashi (Tohoku University, Sendai, Japan). Nrf2-deficient mice were purchased from RIKEN Bioresource Center through the National BioResource Project (Ibaraki, Japan). The mice were caged individually in a room maintained at 18–24 °C with 40–70% relative humidity and a 12-h light/dark cycle. The mice were allowed free access to drinking water and fed the rodent diet CE-2 (Nihon Clea, Tokyo, Japan) during their 1-week acclimatization period. Mouse maintenance and all subsequent experimental procedures were performed in accordance with the National Institutes of Health (NIH) guidelines for the use of experimental animals. All programs were approved [M2021-127, 128, 130, and 132] by the Animal Care Committee of the Kyoto Prefectural University of Medicine (Kyoto, Japan).

### 2.2. Materials

Tricarbonylchloro(glycinato)ruthenium (II) and CORM-3 (mol wt 294.61) were provided as gifts by Prof. Gediminas Cepinskas (University of Western Ontario, London, Canada). Tin protoporphyrin IX (SnPP), an HO-1 inhibitor, was purchased from Santa Cruz Biotechnology (Dallas, TX, USA). Endothelial cell basal medium-2 and EGM-2-MV were purchased from Lonza (Basel, Switzerland). Fetal calf serum, penicillin, and streptomycin were purchased from Sigma-Aldrich (Tokyo, Japan).

### 2.3. Experimental Design

WT, Bach1-deficient, and Nrf2-deficient mice were divided into two groups: (1) sham-operated mice (sham group) (*n* = 7) and (2) intestinal IR-induced mice (IR group) (*n* = 7). The mice were fasted for 12 h prior to the experiment. Mice were anesthetized via intraperitoneal injection of xylazine (10 mg/kg) and ketamine (50 mg/kg). The superior mesenteric arteries were clamped at their bases using a clip, as described in our previous report [16]. After 45 min of occlusion, the clip was removed, and the small intestine was reperfused for 4 h. In another set of experiments, CORM-3 (10 mg/kg) [17,18] diluted in phosphate-buffered saline (PBS) (*n* = 7) was administered intraperitoneally (i.p.) to WT mice 1 h before the sham operation or intestinal IR. SnPP (5 mg/kg) (*n* = 5) was administered intraperitoneally (i.p.) to IR-induced Bach1-deficient mice 1 h before intestinal IR. Sham mice were injected with vehicle (PBS). The mice were euthanized using xylazine and ketamine.

### 2.4. Analysis Methods in Mice

#### 2.4.1. Luminal Protein and Hemoglobin

A segment of the ileum was excised and subjected to measurement of luminal protein and hemoglobin levels. Saline solution (5 mL) was injected into the intestinal loop from the oral side to collect the luminal contents from the other side. Protein and hemoglobin levels in the intestinal lumen were measured to evaluate intestinal injury. Hemoglobin levels, which reflect intestinal bleeding, were measured using a Hemoglobin-Test-Wako kit (Wako Pure Chemical Industries, Osaka, Japan), and protein concentration was measured using a Bio-Rad Protein Assay kit (Bio-Rad Laboratories, Hercules, CA, USA), according to the manufacturer’s protocol. The concentrations of tumor necrosis factor (TNF)-α and keratinocyte chemoattractant (KC) in the supernatant of mucosal homogenates were determined using an enzyme-linked immunosorbent assay (ELISA) kit (eBioscience Inc., San Diego, CA, USA), according to the manufacturer’s instructions. After color development, optical densities were measured at 450 nm using a microplate reader (Spectramax M2; Molecular Devices Corp., Sunnyvale, CA, USA).

#### 2.4.2. Histologic Examination of Intestinal Tissues

Small intestine samples were gently rinsed with normal saline solution, fixed in formalin (Wako Pure Chemical Industries), and embedded in paraffin (Wako Pure Chemical Industries). The sections were deparaffinized, hydrated, and stained with hematoxylin and eosin. Histopathological findings were evaluated under a microscope (BX50; Olympus, Tokyo, Japan).

#### 2.4.3. Measurement of Myeloperoxidase (MPO) Activity

Tissue-associated MPO activity was measured in intestinal tissues as an index of neutrophil accumulation using a modified method reported by Grisham et al. [9]. Intestinal mucosal samples were homogenized in 1 mL of 10 mM potassium phosphate buffer (pH 7.8) containing 30 mM KCl using a Teflon Potter-Elvehjem homogenizer. The mashed intestinal mucosal samples were centrifuged at 20,000× *g* for 15 min at 4 °C. The resulting pellet was re-homogenized in 0.3 mL of 50 mM potassium phosphate buffer (pH 5.4) containing 0.5% hexadecyltrimethylammonium bromide. The mixture was then centrifuged at 20,000× *g* for 15 min at 4 °C, and the supernatants were collected. MPO activity was assessed by measuring H_2_O_2_-dependent oxidation of 3,3′,5,5′-tetramethylbenzidine. One unit of enzyme activity was defined as the amount of MPO that changed the absorbance by 1.0/min at 460 nm and 37 °C. The total protein content in the tissue homogenates was measured using a Bio-Rad Protein Assay kit (Bio-Rad Laboratories, K. K., Tokyo, Japan), according to the manufacturer’s protocol.

#### 2.4.4. Analysis of mRNA Expression Levels in the Intestine

The intestinal mRNA expression levels were determined using real-time polymerase chain reaction (PCR). Total RNA was extracted using the acid-guanidinium-phenol-chloroform method (Isogen Kit; Nippon Gene, Tokyo, Japan). Extracted RNA was stored at −80 °C until use in real-time PCR. A total of 1 μg of RNA was reverse-transcribed to first-strand complementary DNA (cDNA) using a High Capacity cDNA Reverse Transcription kit (Applied Biosystems, Foster City, CA, USA). Real-time PCR was carried out on a 7300 Real-Time PCR system (Applied Biosystems, Foster City, CA, USA) using the DNA-binding dye SYBR Green to detect the PCR products using the following primers: *tnf-α* (NM_013693), sense 5′-ATCCGCGACGTGGAACTG-3′, and antisense 5′-ACCGCCTGGAGTTCTGGAA-3′; *kc* (NM_008176), sense 5′-TGTCAGTGCCTGCAGACCAT-3′, and antisense 5′-CCTGAGGGCAACACCTTCA-3′; intercellular adhesion molecule 1 (*icam-1*) (NM_010493.3), sense 5′-CCGCAGGTCCAATTCACT-3′, and antisense 5′-CAGAGCGGCAGAGCAAAAG-3′; *e-selectin* (M87862.1), sense 5′-CCCTGCCCACGGTATCAG-3′, and antisense 5′-ACGTGCATGTCGTGTTCCAT-3′; *ho-1* (NM_010442.2), sense 5′-CCTCACTGGCAGGAAATCATC-3′, and antisense 5′-CCTCGTGGAGACGCTTTACATA-3′; and *β-actin* (NM_007393.5), sense 5′-TATCCACCTTCCAGCAGATGT-3′, and antisense 5′-AGCTCAGTAACAGTCCGCCTA-3′. Gene expression was normalized to that of β-*actin* as an internal control.

#### 2.4.5. Sodium Dodecyl Sulfate-Polyacrylamide Gel Electrophoresis (SDS-PAGE) and Western Blotting

Total protein from the colonic mucosa was mixed with SDS sample buffer. The samples were subjected to 12% SDS-PAGE and blotted onto a nitrocellulose membrane. The membrane was probed with a rabbit polyclonal anti-HO-1 antibody (SPA-895, 1:1000 dilution in TBS-T; Enzo Life Sciences Inc., Farmingdale, NY, USA) and goat polyclonal anti-Actin (Ab8229, 1:1000 dilution; Abcam, Cambridge, MA, USA) at room temperature for 1 h. After 3 washes with TBS-T, the membrane was incubated with anti-rabbit IgG-HRP and anti-mouse IgG-HRP (1:10,000; Thermo Fisher Scientific, Waltham, MA, USA) at room temperature for 1 h. Immunoreactive proteins were visualized using the ECL Prime western blotting Detection Reagent (GE Healthcare, Buckinghamshire, UK). The band intensities were determined using ImageJ software (version 1.51) (National Institutes of Health, Bethesda, MD, USA).

#### 2.4.6. Preparations of Nuclear Extracts and Electrophoretic Mobility Shift Assay (EMSA)

Nuclear proteins from whole tissues were extracted as previously described [19]. Briefly, frozen tissues were homogenized in PBS containing the following protease inhibitors: 2 mM 4-(2-aminoethyl) benzenesulfonyl fluoride hydrochloride, 1 mM E-64, and 10 μg/mL each of pepstatin A, bestatin, leupeptin, and aprotinin. The homogenate was centrifuged at 3000× *g* for 10 min, and the pellet was resuspended in 2 mL of buffer A (0.3 mol/L sucrose, 5 mmol/L dithiothreitol, 5 mM MgCl_2_, 10 mM Tris-HCl, and 0.1% Triton X-405) and further homogenized using a Dounce homogenizer. After filtration through a 100-μm nylon mesh, the obtained suspension was centrifuged at 1000× *g* for 5 min at 4 °C. The pellet (nuclei) was washed in buffer A without 0.1% Triton X-405 and centrifuged (1000× *g* for 5 min at 4 °C). The nuclei were extracted on ice for 30 min in 60 µL of buffer containing 20 mmol/L HEPES, 0.75 mmol/L spermidine, 0.15 mmol/L spermine, 0.2 mmol/L ethylenediaminetetraacetic acid, 2 mmol/L dithiothreitol, 20% glycerol, and 1 mmol/L phenylmethylsulfonyl fluoride (4 °C) in the presence of 0.4 mol/L NaCl. Finally, the samples were centrifuged for 10 min at 21,000× *g* at 4 °C, and the supernatants were collected and stored at −80 °C as the nuclear protein fraction. For EMSA, 5 μg of total nuclear protein was incubated with 1.0 pmol of double-stranded γ-[32P]-ATP end-labeled oligonucleotides containing consensus binding sequences for NF-κB and electrophoresed on 4% PAG under non-denaturing conditions. Subsequently, the gels were dried and exposed to an X-ray film (Kodak Japan Ltd., Tokyo, Japan) for 2–4 h at −80 °C. Band intensities were determined using ImageJ software (version 1.51).

### 2.5. Various Analysis Methods in Cells

#### 2.5.1. Mesenteric Microvessel Endothelial Cells (MECs)

Mesenteric MECs were isolated as previously described [20]. Briefly, the mesentery was minced, digested (300 U/mL collagenase II and 0.6 U/mL dispase II in HBSS) for 35 min at 37 °C, and filtered through a 100-µm nylon mesh. The filtrate was washed and subsequently co-incubated with rat anti-mouse PECAM-1 antibody (BD Biosciences, Franklin Lakes, NJ, USA) at 4 °C for 30 min. The suspension was washed and co-incubated with microbeads coated with sheep anti-rat IgG antibody for 20 min, and the cells were captured using Dynal Magnet. Captured MMECs were seeded in fibronectin-coated flasks and cultured in endothelial cell basal medium-2 supplemented with EGM-2-MV. The MMEC cultures were >85% pure, as assessed by 1,1′-dioctadecyl-3,3,3′,3′-tetramethylindocarbocyanine (Dil)-acetylated low-density lipoprotein uptake and E-selectin expression in response to lipopolysaccharide. First- and second-passage cells were used for experiments. PMNs were isolated from the bone marrow of adult mice. The long bones of the hind legs were removed and flushed with ice-cold Ca^2+^- and Mg^2+^-free HBSS. The obtained marrow was centrifuged, resuspended, and subjected to a Percoll step gradient (Sigma-Aldrich, Tokyo, Japan). Cells were removed from the neutrophil-enriched fraction. This procedure yielded 4–5 million white blood cells, 95% of which were adult PMNs.

#### 2.5.2. PMN Adhesion Assay

MMECs were isolated from WT and Bach1-deficient mice and stimulated with TNF-α (10 ng/mL) for 4 h. For the adhesion assay, 5 × 10^7^ PMN/mL cells were radiolabeled with 50 μCi Na^51^CrO_4_ in PBS for 60 min at 37 °C. Radiolabeled PMNs (5 × 10^5^/well) were added to MMEC monolayers grown in a 48-well plate (Falcon) and, 30 min later, the percentage of PMNs that remained adherent after a wash procedure was quantified as follows: %PMN adherence = lysate (cpm)/[supernatant (cpm) + wash (cpm) + lysate (cpm)], where cpm is counts per minute [21].

### 2.6. Statistical Analyses

Data are expressed as mean ± standard error of the mean (SEM). The overall differences between groups were determined using one-way analysis of variance (ANOVA). Differences between individual groups were determined using Tukey’s multiple comparison test when one-way ANOVA indicated a significant difference. Target gene copy numbers were estimated using real-time PCR, and the concentration of organic acids was analyzed using the Steel–Dwass method after the Friedman test. Statistical significance was set at *p* < 0.05. All analyses were performed using GraphPad Prism 7 and 9 (GraphPad Software, San Diego, CA, USA).

## 3. Results

### 3.1. Effect of Bach1 Deficiency on the Inflammatory Response in Intestinal IR Injury

**Figure 1:**
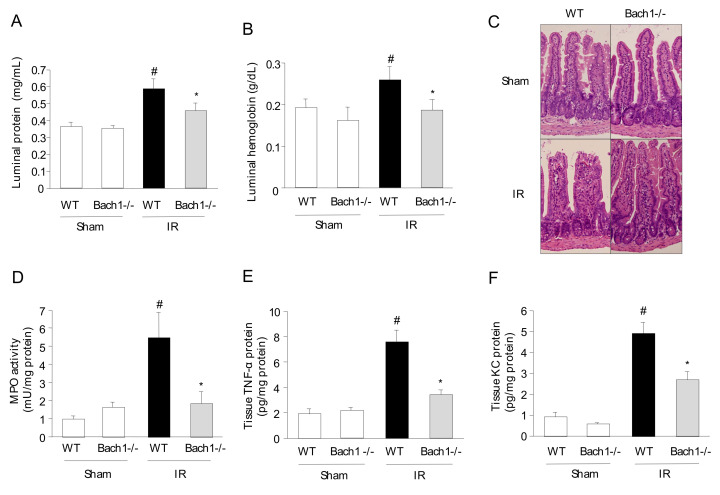
Effect of BTB and CNC homolog 1 (Bach1) deficiency on the inflammatory response in intestinal ischemia-reperfusion (IR) injury. (**A**) Luminal protein (a Bio-Rad Protein Assay kit) (*n* = 7) and (**B**) luminal hemoglobin (a Hemoglobin-Test-Wako kit) (*n* = 7). (**C**) Representative histological appearance in the intestinal tissues (hematoxylin and eosin stain; ×20). (**D**) Myeloperoxidase (MPO) activity in the intestinal mucosa (MPO assay by Grisham et al. [9]) (*n* = 7). (**E**) Tissue tumor necrosis factor (TNF)-α protein and (**F**) tissue keratinocyte chemoattractant (KC) protein (eBioscience ELISA kit) levels (*n* = 7). Sham operation or intestinal IR injury was induced in the wild-type and Bach1-deficient mice. Each value represents the mean ± standard error of the mean (SEM). # *p* < 0.05 compared with sham-operated wild-type (WT) mice; * *p* < 0.05 compared with IR-induced WT mice.

To determine whether Bach1 deficiency interferes with IR-induced inflammatory responses in the intestine, the small intestine (jejunum) obtained from each group of mice was assessed for luminal protein and liminal hemoglobin. Liminal protein and hemoglobin levels were significantly upregulated in the intestines of IR-induced WT mice compared with those of sham-operated WT mice (Figure 1A,B). The increase in luminal protein and hemoglobin levels was effectively reduced in the intestines of the IR-induced Bach1-deficient mice. PMN accumulation in the jejunum after intestinal IR was also evaluated. Figure 1C shows the typical histological appearance of the small intestinal tissues of each group. Large areas of damaged epithelial villi, dilated capillaries, hemorrhage, and inflammatory cell infiltration were observed in the intestine of IR-induced WT mice. In contrast, these morphological impairments were attenuated in the intestines of the IR-induced Bach1-deficient mice. The induction of intestinal IR resulted in an increase in PMN accumulation in the small intestine, as assessed by MPO activity (Figure 1D). Bach1 deficiency significantly reduced PMN infiltration into the IR-induced small intestine. TNF-α and KC protein expression was significantly upregulated in the IR induced small intestine of WT mice (Figure 1E,F). Interestingly, the elevated levels of mucosal TNF-α and KC proteins were significantly reduced in the IR-induced small intestine of Bach1-deficient mice. These results clearly demonstrated that Bach1 deficiency is associated with a decrease in inflammatory responses in intestinal IR injury.

### 3.2. Effect of Bach1 Deficiency on HO-1 Upregulation in Intestinal IR Injury

**Figure 2:**
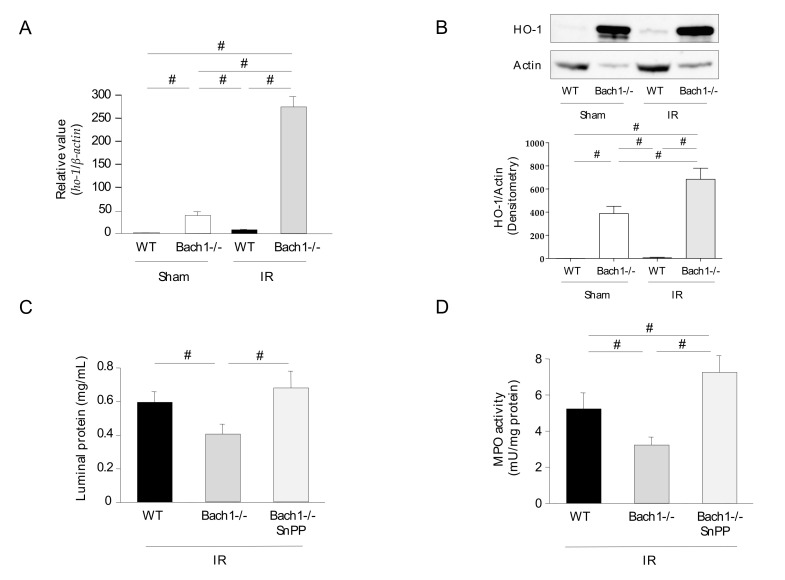
Effect of Bach1 deficiency on heme oxygenase (HO)-1 expression in intestinal IR injury. (**A**) *ho-1* mRNA expression levels (real-time PCR) (*n* = 7), (**B**) HO-1 protein expression levels (western blotting) (*n* = 4), (**C**) Luminal protein expression levels (a Bio-Rad Protein Assay kit) (*n* = 5), and (**D**) MPO activity (*n* = 5). Sham operation or intestinal IR injury was induced in the wild-type and Bach1-deficient mice. Tin protoporphyrin IX (SnPP), an HO-1 inhibitor, was administered to IR-induced Bach1-deficient mice. Each value represents the mean ± SEM. # *p* < 0.05 compared between the selected groups.

The expression of *ho-1* mRNA and protein in the intestines of Bach1-deficient mice was consistently upregulated compared with that in WT mice, as previously reported. These expression levels were further upregulated following intestinal IR (Figure 2A,B). To determine whether Bach1 deficiency attenuates inflammatory responses in intestinal IR injury through HO-1 upregulation, an HO-1 inhibitor (SnPP) was administered to Bach1-deficient mice following intestinal IR. The attenuation of intestinal IR injury (luminal protein and MPO activity) in Bach1-deficient mice was significantly inhibited by the administration of SnPP (Figure 2C,D).

### 3.3. Effects of Bach1 Deficiency on NF-κB Activation, Expression Levels of Adhesion Molecules, and Neutrophil-Endothelial Cell Interaction in Intestinal IR Injury

**Figure 3:**
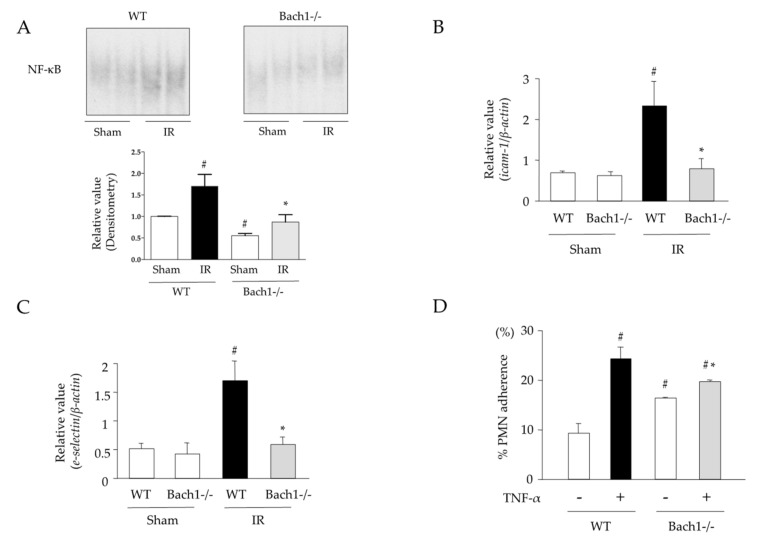
Effects of Bach1 deficiency on NF-κB activation, expression levels of adhesion molecules, and neutrophil-endothelial cell interaction in intestinal IR injury. (**A**) A representative electrophoretic mobility shift assay (EMSA) image and densitometry analysis (*n* = 4–6). (**B**) Intercellular adhesion molecule 1 (*icam-1*) and (**C**) *e-selectin* mRNA expression levels (real-time PCR) (*n* = 7). Sham operation or intestinal IR injury was induced in the wild-type and Bach1-deficient mice. (**D**) Neutrophil adherence rate (PMN adhesion assay) (*n* = 5). Mesenteric microvessel endothelial cells derived from WT or Bach1-deficient mice were used. Each value represents the mean ± SEM. # *p* < 0.05 compared with sham-operated WT mice; * *p* < 0.05 compared with IR-induced WT mice.

The transcription factor NF-κB plays a key role in the initiation of inflammation by inducing the expression of various inflammation-related genes (e.g., TNF-α, IL-1β, and iNOS) [22,23]. The activation of NF-κB is associated with the increased expression of adhesion molecules, such as ICAM-1 and E-selectin [21,24]. Therefore, the activation (nuclear localization) of NF-κB was assessed. Nuclear levels of NF-κB were significantly elevated in the intestines of IR-induced WT mice 1 h after reperfusion (Figure 3A). This activation was significantly lower in the intestines of the IR-induced Bach1-deficient mice. In parallel, the increased expression of adhesion molecules, such as *icam-1* and *e-selectin* mRNA, was significantly lower in the intestines of IR-induced Bach1-deficient mice compared with those of IR-induced WT mice (Figure 3B,C). In another series of experiments, neutrophil-endothelial cell interactions were assessed using MMECs and PMNs. The PMN adhesion rate was significantly increased in TNF-α-stimulated MMECs derived from WT mice. However, the increase in PMN adhesion was significantly attenuated in TNF-α-stimulated MMECs derived from Bach1-deficient mice (Figure 3D).

### 3.4. Effect of Nrf2 Deficiency on the Inflammatory Response in Intestinal IR Injury

**Figure 4:**
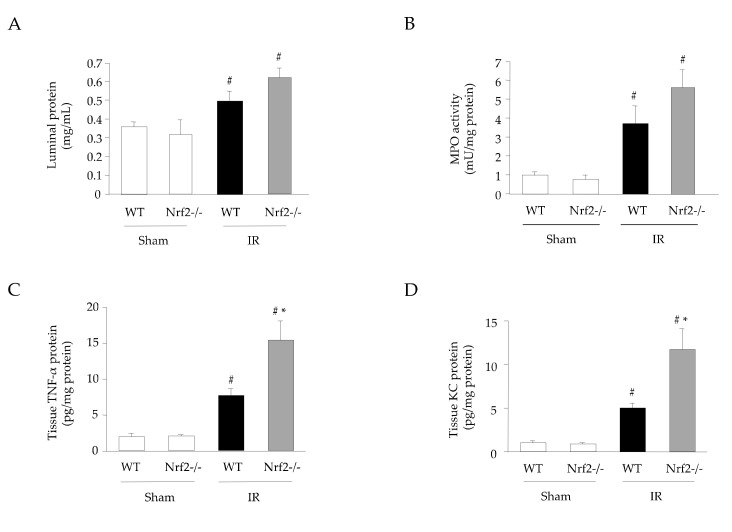
Effect of nuclear factor-erythroid 2-related factor 2 (Nrf2) deficiency on the inflammatory response in intestinal IR injury. (**A**) Luminal protein level (a Bio-Rad Protein Assay kit) (*n* = 7), (**B**) MPO activity (*n* = 7), (**C**) tissue TNF-α protein level, and (**D**) tissue KC protein levels (eBioscience ELISA kit) (*n* = 7). Sham operation or intestinal IR injury was induced in the wild-type and Nrf2-deficient mice. Each value represents the mean ± SEM. # *p* < 0.05 compared with sham-operated WT mice; * *p* < 0.05 compared with IR-induced WT mice.

**Figure 5:**
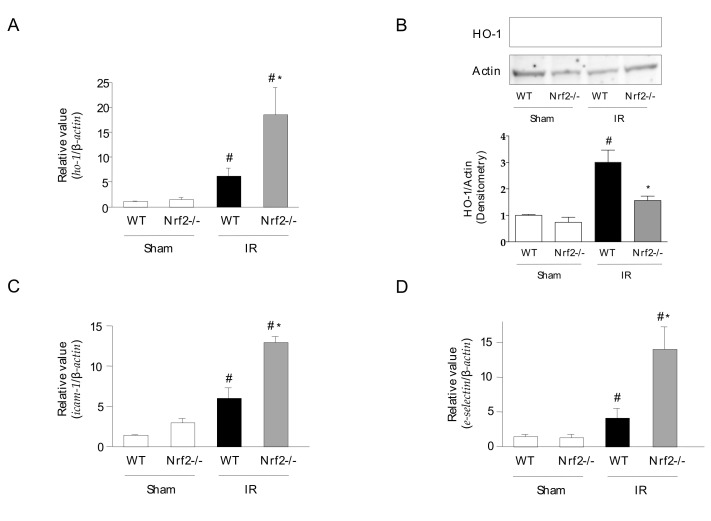
Effects of Nrf2 deficiency on HO-1 expression levels and adhesion molecules in intestinal IR injury. (**A**) *ho-1* mRNA expression levels (real-time PCR) (*n* = 7), (**B**) HO-1 protein expression levels (western blotting) (*n* = 4), (**C**) *icam-1*, and (**D**) *e-selectin* mRNA expression levels (real-time PCR) (*n* = 7). Sham operation or intestinal IR injury was induced in the wild-type and Nrf2-deficient mice. Each value represents the mean ± SEM. # *p* < 0.05 compared with sham-operated WT mice; * *p* < 0.05 compared with IR-induced WT mice.

The effects of Nrf2 deficiency on intestinal IR injury were investigated in Nrf2-deficient mice. Nrf2 is a transcription factor that promotes HO-1 expression. Figure 4 shows that luminal protein, MPO activity, and tissue TNF-α and KC protein levels were significantly augmented in the intestines of IR-induced Nrf2-deficient mice compared with those of WT mice. The expression of *ho-1* mRNA in the intestine of Nrf2-deficient mice was similar to that in WT mice; however, the induction of IR significantly elevated the expression of *ho-1* mRNA in the intestine of Nrf2-deficient mice (Figure 5A). In contrast, the expression of HO-1 protein was significantly decreased in the intestine of IR-induced Nrf2 mice compared with that in WT mice (Figure 5B). Additionally, the mRNA expression of NF-κB-related adhesion molecules *icam-1* and *e-selectin* was significantly higher in the intestines of IR-induced Nrf2-deficient mice than in WT mice (Figure 5C,D).

### 3.5. Effect of CO Derived from CORM-3 on the Inflammatory Response in Intestinal IR Injury

**Figure 6:**
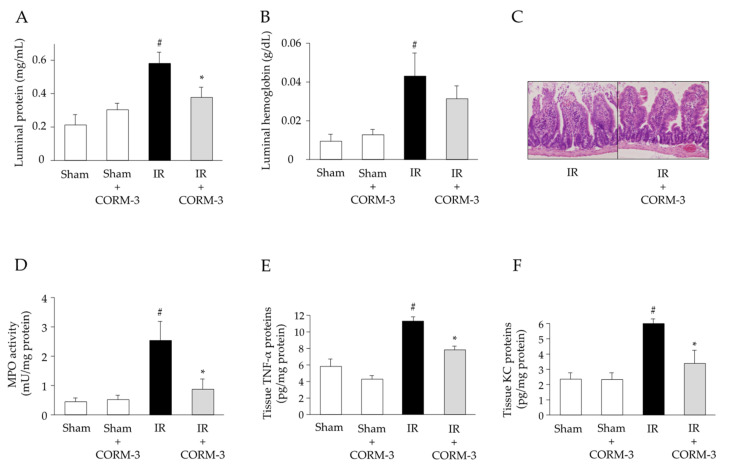
Effect of carbon monoxide-releasing molecule (CORM)-3 on the inflammatory response in intestinal IR injury. (**A**) Luminal protein (a Bio-Rad Protein Assay kit) (*n* = 7) and (**B**) luminal hemoglobin levels (a Hemoglobin-Test-Wako kit) (*n* = 7). (**C**) Representative histological appearance in the intestinal tissues (hematoxylin and eosin stain; ×20). (**D**) MPO activity (MPO assay) (*n* = 7), (**E**) tissue TNF-α protein levels, and (**F**) tissue KC protein levels (eBioscience ELISA) (*n* = 7). Sham operation or intestinal IR injury with or without CORM-3 administration was induced in the wild-type mice. Each value represents the mean ± SEM. # *p* < 0.05 compared with sham-operated WT mice; * *p* < 0.05 compared with IR-induced WT mice.

To determine whether CORM-3-released CO could interfere with IR-induced inflammatory responses in the intestine, luminal protein and hemoglobin levels were assessed in the small intestine obtained from each group of mice. Luminal protein and hemoglobin levels were significantly increased in the IR-induced intestine (Figure 6A,B). CORM-3 administration significantly reduced the increased levels of luminal proteins but not those of luminal hemoglobin. Large areas of damaged epithelial villi, dilated capillaries, hemorrhage, and inflammatory cell infiltration were observed in the intestines of IR-induced WT mice, which were attenuated by the administration of CORM-3 (Figure 6C). CORM-3 administration significantly reduced the marked increase in PMN accumulation in the intestines of IR-induced WT mice (Figure 6D). TNF-α and KC protein expression levels were significantly upregulated in the IR-induced small intestine of WT mice (Figure 6E,F). Elevated levels of mucosal TNF-α and KC proteins were significantly decreased by CORM-3 treatment.

### 3.6. Effects of CO Derived from CORM-3 on NF-κB Activation and Expression Levels of Adhesion Molecules in Intestinal IR Injury

**Figure 7:**
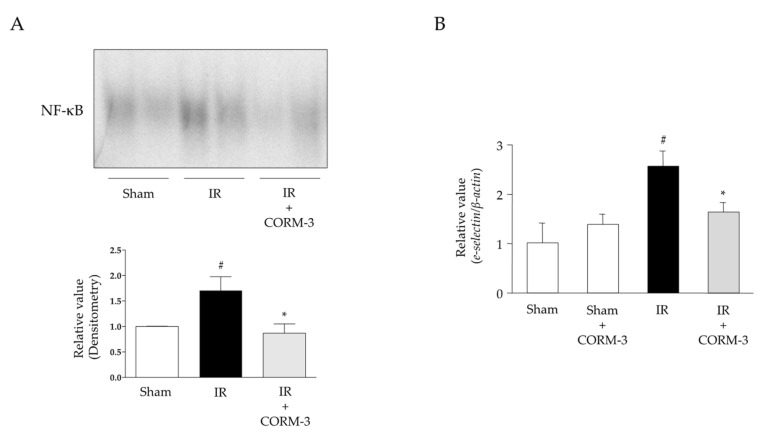
Effects of CORM-3 on nuclear factor (NF)-κB activation and expression levels of downstream adhesion molecule E-selectin in intestinal IR injury. (**A**) A representative electrophoretic mobility shift assay (EMSA) image and densitometry analysis (*n* = 4–6). (**B**) *e-selectin* mRNA expression (real-time PCR) (*n* = 7). Sham operation or intestinal IR injury with or without CORM-3 administration was induced in the wild-type mice. Each value represents the mean ± SEM. # *p* < 0.05 compared with sham-operated WT mice; * *p* < 0.05 compared with IR-induced WT mice.

NF-κB activation was significantly increased in the intestines of IR-induced WT mice compared with that in sham-operated WT mice (Figure 7A). The administration of CORM-3 significantly reduced IR-induced NF-κB activation. Furthermore, the increased expression of NF-κB-regulated E-selectin was significantly suppressed by CORM-3 administration (Figure 7B).

## 4. Discussion

Our results indicated that luminal inflammatory markers, such as luminal protein and hemoglobin levels, tissue levels of TNF-α and KC, and subsequent PMN accumulation, were significantly elevated in the intestines of WT mice with IR. These changes were significantly attenuated in Bach1-deficient mice or mice treated with CORM-3 and were exacerbated in Nrf2-deficient mice. In addition, treatment with an HO-1 inhibitor reversed the attenuation of IR-induced injury in Bach1-deficient mice. Bach1 deficiency and treatment with CORM-3 resulted in the downregulation of NF-κB activation and suppression of downstream adhesion molecules in the intestinal mucosa.

Many reports have confirmed the antioxidant, anti-inflammatory, and cytoprotective effects of HO-1 inducers, which subsequently produce CO and CO inducers in small intestinal injuries induced by IR [15]. In these studies, HO-1 was upregulated by various treatments, including hypothermia, hypertonic saline, hyperthermia, cobalt protoporphyrin, ischemic preconditioning, octreotide, hydrogen sulfide, hydrogen, sulforaphane, hemin, bilirubin, and glutamine, all of which protect against intestinal IR injury. With regard to the transcription factor HO-1, sulforaphane promotes the activation of Nrf2/HO-1 signaling in intestinal IR injury [25], and hypothermia prevents NF-κB activation in intestinal IR injury [26]. In addition to sulforaphane, higenamine [27], salvianolic acid A [28], dimethyl fumarate (DMF) [29], and sesamin [30] have recently been reported to protect against intestinal IR injury via Nrf2/HO-1. Interestingly, DMF has previously been used for the treatment of psoriasis in Germany, and DMF (>0.1 ppm) is now used in the European Union. Various Nrf2 activators have been developed. In this study, Nrf2 deficiency exacerbated inflammatory responses in intestinal IR injury, which may be mediated by the inhibition of downstream antioxidant enzymes, including HO-1. *ho-1* mRNA was upregulated in IR-induced Nrf2-deficient mice compared with that in IR-induced WT mice in this study. However, HO-1 protein levels were downregulated in IR-induced Nrf2-deficient mice compared with that in IR-induced WT mice. The HO-1 protein levels are inconsistent with the *ho-1* mRNA levels because we speculate that mRNA and protein levels are both measured at the same time point and that there is the potential for a posttranslational modification or degradation of HO-1. Further examinations are needed. Ultimately, the data of HO-1 protein expression suggests that HO-1 expression is mainly regulated by Nrf2 compared with other transcription factors, such as NF-κB. On the contrary, a recent report has indicated that NF-κB is a key regulator of oxidative stress-induced necrosis by suppressing the Nrf2-ARE antioxidant pathway [31]. Further investigations of the regulation of Nrf2 and NF-κB are required.

There are no reports on the effect of Bach1 on intestinal IR injury. This study is the first to discuss the effect of Bach1 on intestinal I_R injury. In contrast, the effects of Bach1 in myocardial, cerebral, and liver IR injuries have already been investigated. Micro RNA-30c-5p protects against myocardial IR injury by regulating Bach1/Nrf2 [32]. Bach1 is downregulated by HO-1 and NQO1, which play important roles in protection against cerebral IR injury [33]. MicroRNA (miR-27a-5p) regulates liver IR injury in mice by targeting Bach1 [34]. These reports demonstrate that the downregulation of Bach1 may be a pivotal target for IR injury, suggesting that a Bach1 inhibitor could be developed for the treatment of intestinal IR injury.

CO itself functions as a signaling molecule that exerts significant cytoprotection because of its anti-inflammatory, vasodilating, and anti-apoptotic properties [35]. CO inhalation is a straightforward delivery method for HO systems; however, excessive CO is toxic and has the important problem of increasing COHb levels. Therefore, alternative methods of delivering CO need to be developed, such as the ex vivo application of CO gas. Cold storage in a preservation solution bubbled with 5% CO has been shown to prevent transplant-induced IR injury [36], clearly suggesting a clinical application of CO in intestinal transplantation. Another way of delivering CO is CORM, which does not elevate COHb levels. Our previous study indicated that CORM-2-released CO confers anti-inflammatory effects on IR-induced intestinal injury in mice by interfering with NF-κB activation and subsequent upregulation of the vascular pro-adhesive phenotype [16]. A new type of CORM will lay the path for a new age in the field of CO research. In this study, a water-soluble CORM (CORM-3) demonstrated anti-inflammatory effects against IR-induced intestinal injury in mice. However, CORM-2 and CORM-3 contained heavy metals in their central parts. Therefore, a new type of CORM without heavy metals, such as CORM-A1 and CORM-401, was developed for future research. Interestingly, a recent report indicated that the luminal application of CORM-3 mitigates IR injury in rats following intestinal transplantation [37]. Therefore, new CO delivery systems should be continuously investigated.

Further studies are required to elucidate the detailed mechanisms involved in this phenomenon. However, targeting HO-1 could show great promise as a therapeutic strategy for intestinal IR injury.

## 5. Conclusions

Our study clearly demonstrates that Bach1 deficiency and CORM 3-released CO significantly attenuate the pro-inflammatory response elicited by IR in the intestine, whereas Nrf2 deficiency exacerbates it via the downregulation of NF-κB activation and suppression of the vascular endothelial cell pro-adhesive phenotype, suggesting that Bach1 inhibitors, Nrf2 inducers, or CO-releasing compounds will be beneficial in treating IR-induced inflammation.

## Data Availability

The data presented in this study are available in this article.
